# The impact of using dexmedetomidine during surgery on the postoperative survival of cancer patients: a meta-analysis and systematic review of real-world studies

**DOI:** 10.3389/fphar.2026.1906464

**Published:** 2026-08-18

**Authors:** Liangqiong Zhu, Ying Liu, Wenlian Mou, Zhifang Luo

**Affiliations:** 1 Operating Room, Chongqing Traditional Chinese Medicine Hospital, Chongqing, China; 2 Department of Anesthesiology, The First Affiliated Hospital of Chongqing University of Chinese Medicine, Chongqing, China; 3 Department of General Surgery, Chongqing Traditional Chinese Medicine Hospital, Chongqing, China

**Keywords:** cancer, dexmedetomidine, meta-analysis, real-world study, surgery, survival, systematic review

## Abstract

**Background:**

Perioperative stress and immunosuppression may influence tumor recurrence and long-term survival in cancer patients. Dexmedetomidine, a widely used anesthetic adjunct, remains controversial regarding its impact on postoperative prognosis. Some studies suggest it may attenuate immunosuppression, whereas others point to a potential tumor-promoting effect. This meta-analysis aimed to synthesize evidence from real-world studies and evaluate the association between intraoperative dexmedetomidine use and post-surgical survival in cancer patients.

**Methods:**

We systematically searched PubMed, Embase, Cochrane Library, Web of Science, China National Knowledge Infrastructure (CNKI), Chinese Biomedical Literature Database, and Wanfang Database from inception to April 2026 for real-world observational studies examining the association between intraoperative dexmedetomidine use and postoperative survival in cancer patients. The primary outcomes were overall survival (OS) and recurrence-free survival (RFS). Two reviewers independently screened studies, extracted data, and assessed risk of bias. Hazard ratios (HRs) with 95% confidence intervals (CIs) were pooled for survival endpoints. Sensitivity analyses tested the robustness of the results, and subgroup analyses were performed according to study characteristics. Publication bias was evaluated using funnel plots, Begg’s test, and Egger’s test. All statistical analyses were conducted with Stata 15.0.

**Results:**

Eight studies comprising 4,613 patients were included. The meta-analysis revealed no statistically significant association between intraoperative dexmedetomidine use and postoperative OS (HR = 0.95, 95% CI (0.72, 1.24), *P* = 0.685) or RFS (HR = 0.85, 95% CI (0.61, 1.20), *P* = 0.363). Subgroup analyses suggested that dexmedetomidine might improve OS in colorectal cancer patients, but no such difference was observed in renal cell carcinoma or other tumor types. Across different sample sizes and geographic regions, dexmedetomidine use was not significantly associated with OS.

**Conclusion:**

This meta-analysis of real-world evidence indicates that intraoperative dexmedetomidine use is not statistically significantly associated with OS and RFS in cancer patients. Although the subgroup finding for colorectal cancer hints at a possible survival benefits, the overall evidence does not support routine dexmedetomidine administration for improving long-term oncological outcomes. Given the limited number of included studies and potential confounding factors, causal inferences should be avoided. Large-scale, prospective real-world studies are warranted to confirm these findings.

**Systematic Review Registration:**

https://www.crd.york.ac.uk/PROSPERO/view/CRD420261370773.

## Introduction

Surgical resection remains the cornerstone of curative treatment for most patients with solid tumors (Raoof et al., 2023). Nevertheless, a substantial proportion of patients still develop recurrence and metastasis despite undergoing radical resection (Choi and Hwang, 2024). This phenomenon is closely linked to perioperative stress responses, inflammatory statesand immunosuppression (Bezu et al., 2024; Teng et al., 2025; Tang et al., 2020). Anesthetic agents and adjuvants may modulate these processes, thereby influencing the fate of microscopic residual disease and potentially impacting long-term postoperative survival.

Dexmedetomidine, a highly selective α2-adrenergic receptor agonist (Zhang et al., 2026), has been widely used in anesthesia for cancer surgery owing to its sedative, analgesic, and sympatholytic properties, as well as its opioid-sparing effect (Liu et al., 2024; Tian et al., 2024). However, findings on its impact on tumor biology remain conflicting. Some preclinical studies have suggested that dexmedetomidine can alleviate surgical-stress-induced immunosuppression, thus theoretically inhibiting tumor progression (Xu et al., 2025; Jun et al., 2023). Conversely, others have indicated that it may promote tumor cell proliferation, migration, and invasion through activation of signalling pathways such as HIF-1α and PI3K/Akt (Gu et al., 2025; Chen et al., 2019). Clinically, several small-sample randomized controlled trials and retrospective cohort studies have yielded discordant results (Luo et al., 2023; Hu et al., 2023; Wong et al., 2025; Yang et al., 2023; Zhao et al., 2021); while some reported improved survival (Chen et al., 2025; Ren et al., 2024), others found no significant association or even suggested potential risks (Wong et al., 2025; Yang et al., 2023; Zhao et al., 2021).

Given the challenges of long follow-up periods, poor adherence, and ethical constraints in randomized controlled trials assessing long-term survival endpoints, real-world studies have increasingly become a valuable source of evidence for evaluating the association between perioperative interventions and cancer prognosis. Therefore, we systematically searched both domestic and international databases for real-world studies comparing intraoperative dexmedetomidine use versus non-use, and performed a meta-analysis to comprehensively assess its association with overall survival (OS) and recurrence-free survival (RFS) in cancer patients after surgery. We also explored potential effect modifiers, aiming to provide evidence-based guidance for perioperative anesthetic management.

## Methods

This meta-analysis was performed in accordance with the Preferred Reporting Items for Systematic Reviews and Meta-Analyses (PRISMA) guidelines (Zhu et al., 2025; Page et al., 2021; Zhang et al., 2024). The protocol was registered in PROSPERO (CRD420261370773).

### Search strategy

We systematically searched PubMed, Embase, Cochrane Library, Web of Science, China National Knowledge Infrastructure (CNKI), Chinese Biomedical Literature Database (CBMdisc), and Wanfang Database from inception to April 2026. The search strategy combines MeSH terms with free-text words, using the following terms: “dexmedetomidine,” “cancer,” “malignancy,” “neoplasm,” “surgery,” “prognosis,” “survival,” “overall survival,” and “recurrence-free survival.” We also manually screened the reference lists of included studies for additional relevant literature. The search was restricted to Chinese and English language publications.

### Inclusion and exclusion criteria

Studies were eligible if they met all the following criteria:

1. Population: pathologically confirmed cancer patients undergoing surgical resection;
2. Exposure: intravenous dexmedetomidine administered during surgery (any dose, rate, or duration), either alone or as an adjunct to general anesthesia;
3. Comparison: no intraoperative dexmedetomidine, with patients receiving conventional anesthesia management (e.g., placebo or standard protocol without dexmedetomidine);
4. Outcome: primary outcome–OS; secondary outcome–RFS;
5. Study design: real-world studies, including prospective and retrospective cohort studies.

Exclusion criteria were:

1. Case series, case reports, reviews, or conference abstracts;
2. Animal or cell experiments;
3. Studies that did not report extractable effect sizes (hazard ratio [HR] and its 95% confidence interval [CI]);
4. Duplicate publications.

### Data extraction

Two reviewers independently screened the literature against the eligibility criteria. They first screened titles and abstracts to exclude obviously irrelevant records, then retrieved full texts for further assessment. Disagreements were resolved through discussion. Data were extracted using a pre-designed standardized form, including:

1. Basic study information: first author, publication year, country, study design, and study period;
2. Participant characteristics: tumor type, sample size, age, and sex distribution;
3. Outcome data: HRs with 95% CIs for OS and RFS; we preferentially extracted adjusted HRs from multivariate analyses; if not available, univariate estimates were used;
4. Follow-up: median follow-up time (months);
5. Adjustment variables: confounders adjusted for in multivariate models (e.g., age, sex, ASA classification, tumor stage);
6. Risk-of-bias assessment items.

If both adjusted and unadjusted HRs were reported, we preferentially extracted adjusted estimates. For studies that did not report HR but provided Kaplan–Meier curves, we used Engauge Digitizer software to digitize the curves and compute HRs.

### Risk of bias

Two reviewers independently assessed the risk of bias of included cohort studies using the Newcastle-Ottawa Scale (NOS). The NOS evaluates three domains: selection of study populations (4 items, maximum 4 stars), comparability between groups (1 item, maximum 2 stars), and outcome measurement (3 items, maximum 3 stars), with a total score of 9 stars. Studies were classified as low quality (0–3 stars, high risk of bias), moderate quality (4–6 stars, moderate risk of bias), or high quality (7–9 stars, low risk of bias). Disagreements were resolved by consensus.

### Statistical analysis

We performed meta-analyses using Stata 15.0. For OS and RFS, we pooled HRs with their 95% CIs. Heterogeneity was assessed using the Q test and I^2^ statistic. When substantial heterogeneity was present (I^2^ > 50% and *P* < 0.10), we performed subgroup and sensitivity analyses to explore its sources. Otherwise (*P* ≥ 0.10 and I^2^ ≤ 50%), a fixed-effects model was used; otherwise, a random-effects model was applied.

To investigate potential effect modifiers and sources of heterogeneity, we predefined the following subgroup analyses:

1. Tumor type: colorectal cancer, renal cell carcinoma, and other tumors;
2. Region: Asian studies (including China, Korea, Japan, etc.) versus non-Asian studies (e.g., the United States, Europe);
3. Sample size: >500 cases versus ≤500 cases.

Due to insufficient reporting of dosing details in the original studies, we could not pre-specify subgroup analyses based on administration regimens, and we acknowledge this as a methodological limitation.

We performed sensitivity analyses by sequentially omitting each study and re-calculating the pooled estimates to assess the robustness of our findings. Publication bias was evaluated using funnel plots, Begg’s test, and Egger’s test.

## Results

### Searching results

**FIGURE 1:**
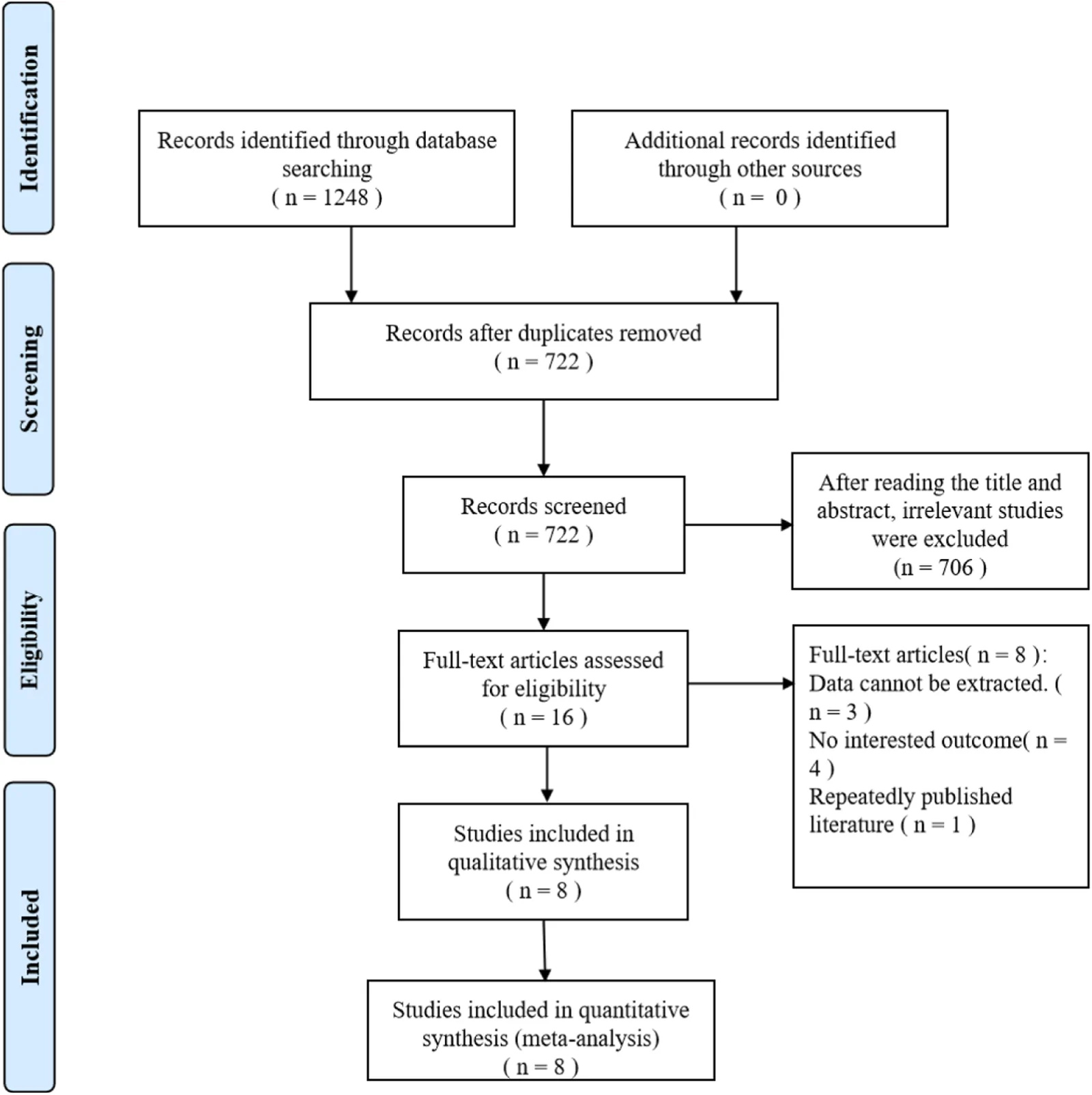
PRISMA flow diagram of the literature selection process.

The initial search yielded 1,248 records. After removing duplicates and performing preliminary screening followed by full-text reassessment, a total of 8 studies (Wong et al., 2025; Yang et al., 2023; Zhao et al., 2021; Chen et al., 2025; Ren et al., 2024; Cata et al., 2017; Owusu-Agyemang et al., 2018; Wang et al., 2023) involving 4,613 cancer surgery patients were finally included (Figure 1).

### Characteristics of eligible studies

Baseline characteristics of the included studies are summarised in Table 1. The eight studies were published from 2017 to 2025, with three conducted in the United States and five in China. The sample size ranged from 76 to 1,770 cases; four studies had >500 participants and four had ≤500. Tumour types comprised colorectal cancer (2 studies), renal cell carcinoma (2 studies), lung cancer (1 study), liver cancer (1 study), breast cancer (1 study), and peritoneal cancer (1 study). All studies reported OS, and four additionally reported RFS. All studies adjusted for potential confounders, including age, sex, ASA classification, tumor stage, surgical approach, and anesthesia duration, etc. The total NOS scores ranged up to 9 stars, with five studies rated as high quality (7–9 stars) and three as moderate quality (4–6 stars) (Table 2). Overall, the methodological quality of the included studies was moderately high.

**TABLE 1:** Baseline characteristics of included studies.

Study	Country	Study design	Time period	Tumor type	Sample size	Age	Sex (Male %)	Outcomes	Follow-up (months)	Adjustment factors
Cata et al., 2017	USA	Retrospective study	From January 2004 to December 2011	Lung cancer	502	65.55	290 (58%)	OS, RFS	58.2, Median follow-up time	Age, gender, ASA, stage of disease, type of surgery, and anesthesia duration
Chen et al., 2025	China	Retrospective study	From January 2011 to December 2018	Colorectal cancer	970	57	627 (64.64%)	OS	NR	Age, gender, BMI, ASA, preoperative HGB, preoperative ALB, Comorbidity, CEA, Tumor location, Tumor differentiation, Pathologic TNM stage, Surgical type, History of abdominal surgery, Duration of operation ≥180, Intraoperative blood loss, Total intraoperative infusion volume, Blood transfusion, Postoperative chemotherapy and postoperative ICU
Owusu-Agyemang et al., 2018	USA	Retrospective study	NR	Peritoneal carcinomatosis	97	12.5	39 (41.94%)	OS	The median OS of the entire study population was 27 months	Extra-abdominal disease, Incomplete cytoreduction, Anesthesia duration
Ren et al., 2024	China	Retrospective study	2014	Colorectal cancer	76	NR	39 (51.32%)	OS, RFS	NR	LDL and ASA classification
Wang et al., 2023	China	Retrospective study	From June 2019 to July 2020	Hepatocellular carcinoma	214	55.35	NR	OS	NR	Drinking, PVTT, Tumor size, Hepatic inflow occlusion, Plasma or RBC transfusion
Wong et al., 2025	USA	Retrospective study	From June 2016 to November 2023	Renal cell carcinoma	1770	59.5	1144 (64.63%)	OS, RFS	NR	Age, gender, ASA, BMI, Tumor staging, Fuhrman score, Surgical procedure, Surgical approach, Anaesthesia time, Length of stay, Recurrence status
Yang et al., 2023	China	Retrospective study	From July 2013 to June 2014	Breast cancer	658	53.3	NR	OS, RFS	NR	Age, BMI, Chemotherapy, Radiotherapy, Hormonal therapy, ER+, PR+, HER2+, Ki67, ASA classification, TMN stage, Type of surgery, Duration of anesthesia, Remifentanil, Sufentanyl, Propofol, Cisatracurium, Inhalable anesthetic, VAS score when leaving PACU, Propensity score
Zhao et al., 2021	China	Retrospective study	From January 2013 to May 2015	Renal cell carcinoma	326	55	90 (72.58%)	OS	NR	Age, gender, ASA, BMI, Duration of anesthesia, Sevoflurane, Fuhrman Grading System, Pathologic TNM stage, Targeted therapy

NR, Not Reported; RFS, recurrence-free survival; OS, overall survival; ASA, American Society of Anaesthesiology; BMI, body mass index; HGB, hemoglobin; ALB, albumin; CEA, carcinoembryonic antigen; ICU, Intensive Care Unit; LDL, Low-density lipoprotein; PVTT, Portal Vein Tumor Thrombus; RBC, Red Blood Cell; PACU, Post-Anesthesia Care Unit; TNM, tumor node metastasis.

**TABLE 2:** The risk of bias in cohort studies.

Reference	Selection score	Comparability score	Outcome score	Total score	Grade
Cata et al., 2017	***	**	***	8	Good
Chen et al., 2025	***	**	***	8	Good
Owusu-Agyemang et al., 2018	***	*	**	6	Moderate
Ren et al., 2024	***	*	**	6	Moderate
Wang et al., 2023	***	**	**	7	Good
Wong et al., 2025	***	**	***	8	Good
Yang et al., 2023	***	**	***	8	Good
Zhao et al., 2021	***	*	**	6	Moderate

### Meta-analysis

**FIGURE 2:**
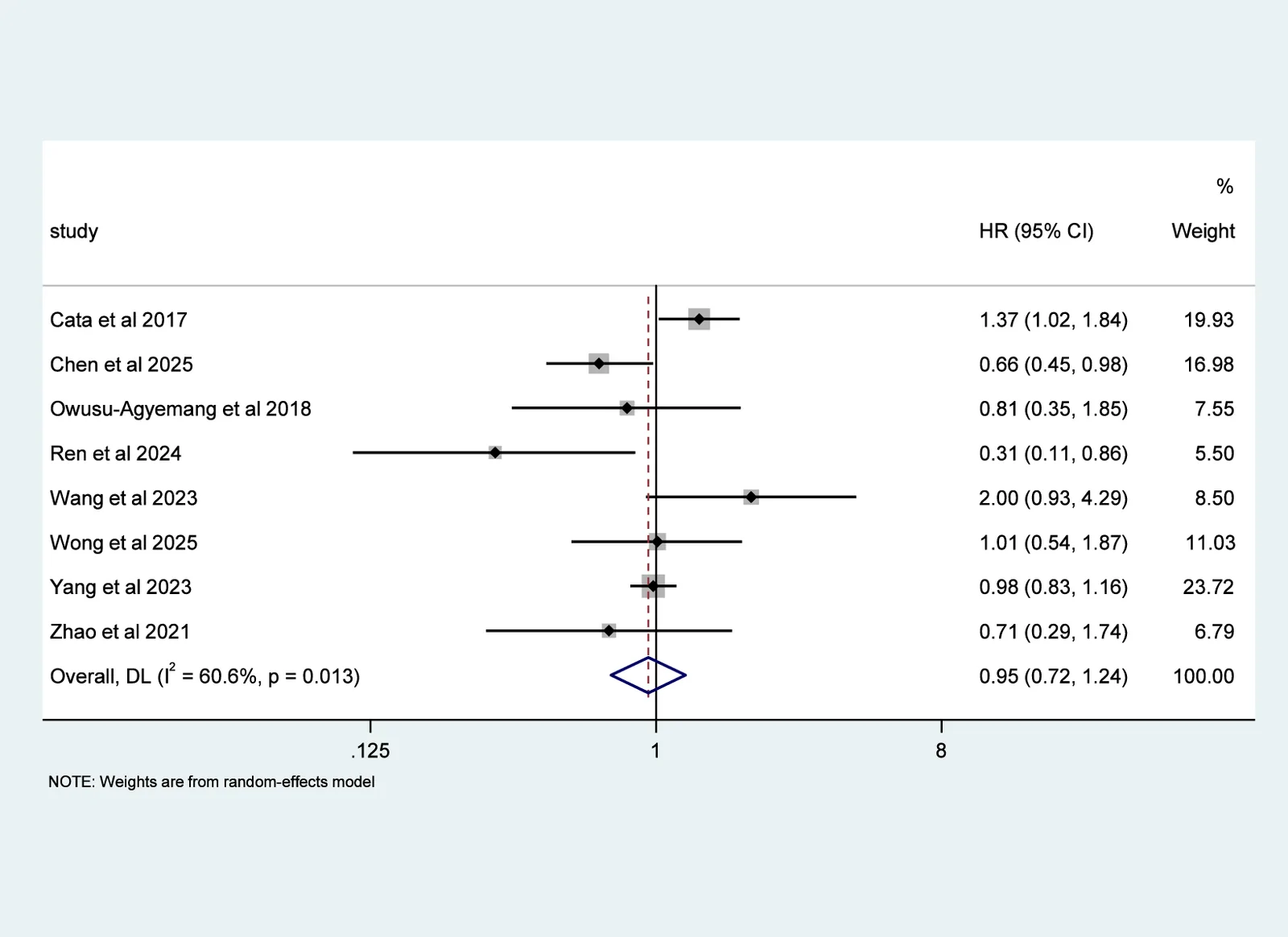
Forest plot of the association between intraoperative use of dexmedetomidine and overall survival in cancer patients after surgery.

All eight studies examined the association between intraoperative dexmedetomidine use and postoperative OS. Heterogeneity was moderate (I^2^ = 60.6%, *P* = 0.013), prompting use of a random-effects model. The pooled analysis showed no statistically significant association between dexmedetomidine use and OS (HR = 0.95, 95% CI:0.72–1.24, *P* = 0.685) (Figure 2).

**FIGURE 3:**
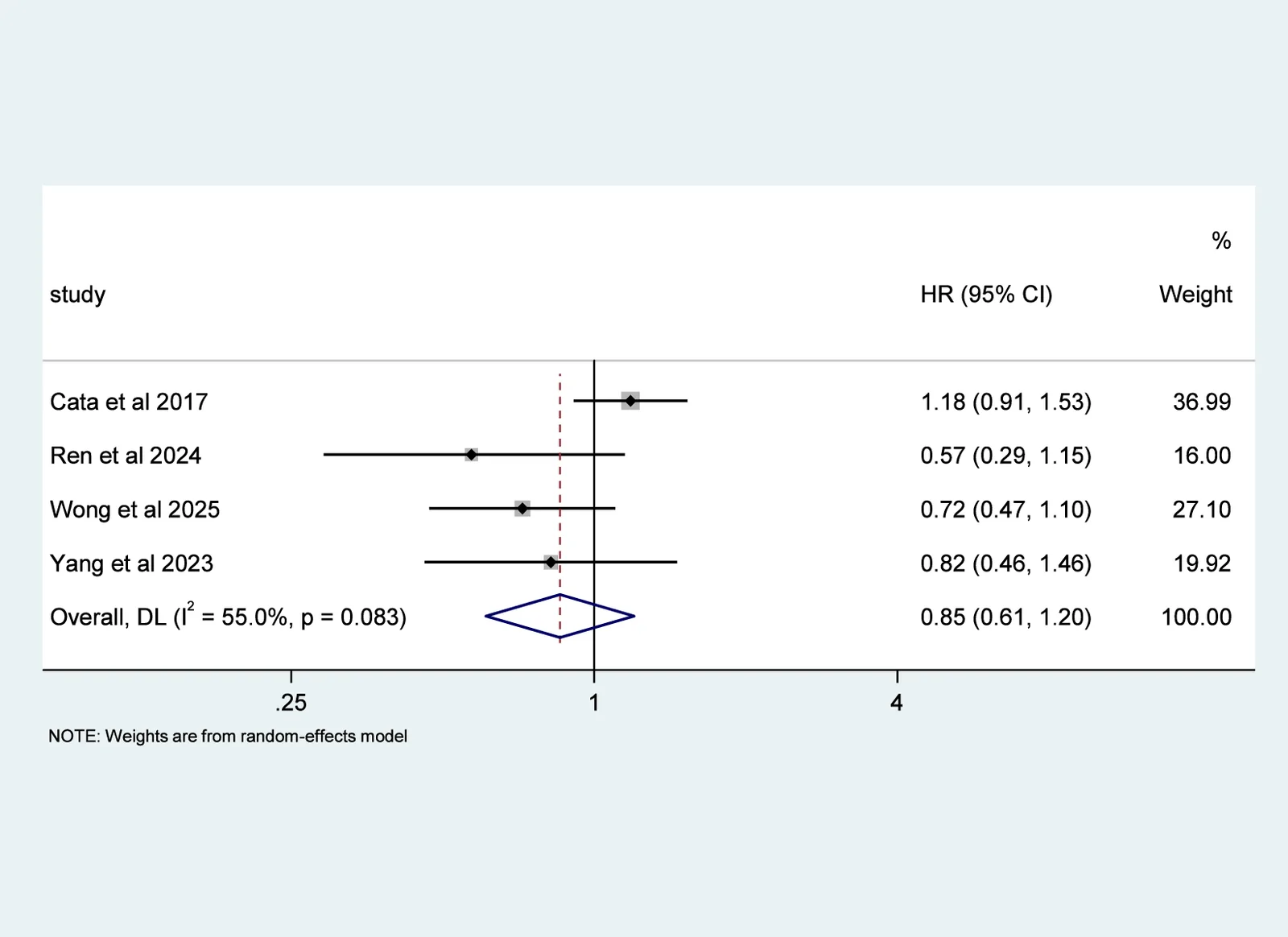
Forest plot of the association between intraoperative use of dexmedetomidine and recurrence-free survival in cancer patients after surgery.

Five studies reported on the association with RFS. Heterogeneity was also moderate (I^2^ = 55.0%, *P* = 0.083), and a random-effects model was applied. No significant association was observed for RFS (HR = 0.85, 95% CI:0.61–1.20, *P* = 0.363) (Figure 3).

### Subgroup analysis

To explore the sources of heterogeneity and the potential effect modifiers, we performed subgroup analyses for OS (Table 3).

1. Tumor type

**TABLE 3:** Subgroup analysis.

Subgroup analysis	Heterogeneity		HR (95% CI)	P Value
	I^2^	P		
Region				
Asian	66.50%	0.018	0.84 (0.56, 1.25)	0.38
Non-Asian	0%	0.394	1.24 (0.96,1.60)	0.097
Tumor type				
Colorectal cancer	45%	0.178	**0.60 (0.42,0.86)**	**0.006**
Renal cell carcinoma	0%	0.526	0.90 (0.54,1.50)	0.688
Other	55.50%	0.08	1.17 (0.87,1.57)	0.296
Sample size				
>500	65.90%	0.032	0.99 (0.75,1.31)	0.932
≤500	65%	0.034	0.81 (0.39,1.70)	0.581

HR, hazard ratio; CI, confidence interval.

Bold indicates the differences were statistically significant.

Colorectal cancer: Two studies pooled data and showed that the use of dexmedetomidine during surgery significantly improved the OS of patients with colorectal cancer (HR = 0.60, 95% CI:0.42–0.86, *P* = 0.006);

Renal cell carcinoma: Two studies pooled data and showed that dexmedetomidine had no significant association with OS in patients with renal cell carcinoma (HR = 0.90, 95% CI:0.54–1.50, *P* = 0.688);

Other tumors: Four studies pooled data and showed that dexmedetomidine was not significantly associated with OS in patients with renal cell carcinoma (HR = 1.17, 95% CI:0.87–1.57, *P* = 0.296).

2. Region

Asian population: Five studies pooled data and showed that dexmedetomidine was not significantly associated with OS in patients with renal cell carcinoma (HR = 0.84, 95% CI:0.56–1.25, *P* = 0.380);

Non-Asian population: Two studies combined showed that dexmedetomidine was not significantly associated with OS in patients with renal cell carcinoma (HR = 1.24, 95% CI:0.96–1.60, *P* = 0.097).

3. Sample size

Sample size >500 cases: Four studies pooled data and showed that dexmedetomidine was not significantly associated with OS in patients with renal cell carcinoma (HR = 0.99, 95% CI:0.75–1.31, *P* = 0.932);

Sample size ≤500 cases: Four studies pooled data and showed that dexmedetomidine was not significantly associated with OS in patients with renal cell carcinoma (HR = 0.81, 95% CI:0.39–1.70, *P* = 0.581).

### Sensitivity analysis

**FIGURE 4:**
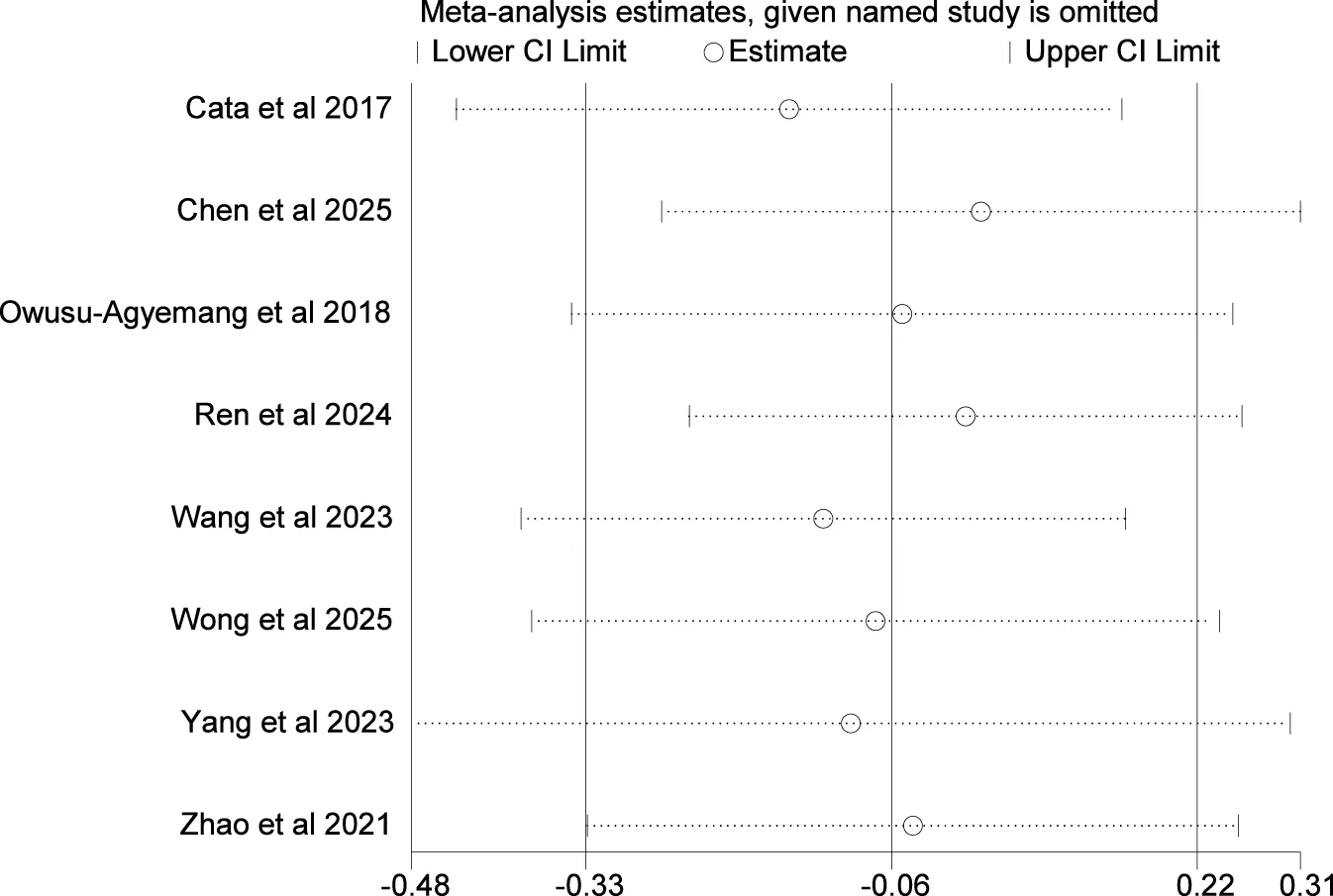
Sensitivity analysis of the association between intraoperative use of dexmedetomidine and overall survival in cancer patients after surgery.

**FIGURE 5:**
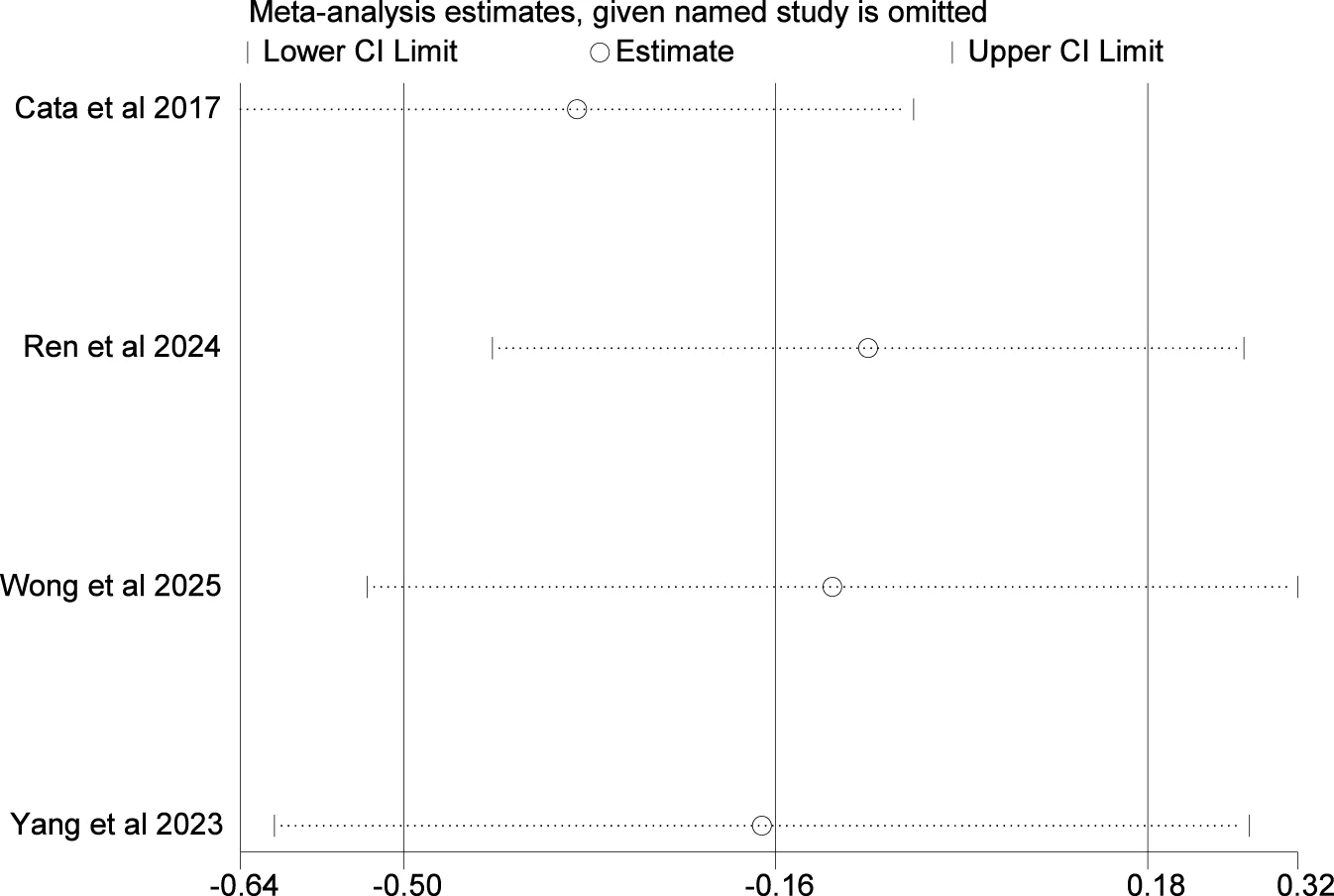
Sensitivity analysis of the association between intraoperative use of dexmedetomidine and recurrence-free survival in cancer patients after surgery.

Sequential exclusion of each individual study did not materially alter the pooled estimates, indicating that the results were not dominated by any single study and were robust (Figures 4, 5).

### Publication bias

**FIGURE 6:**
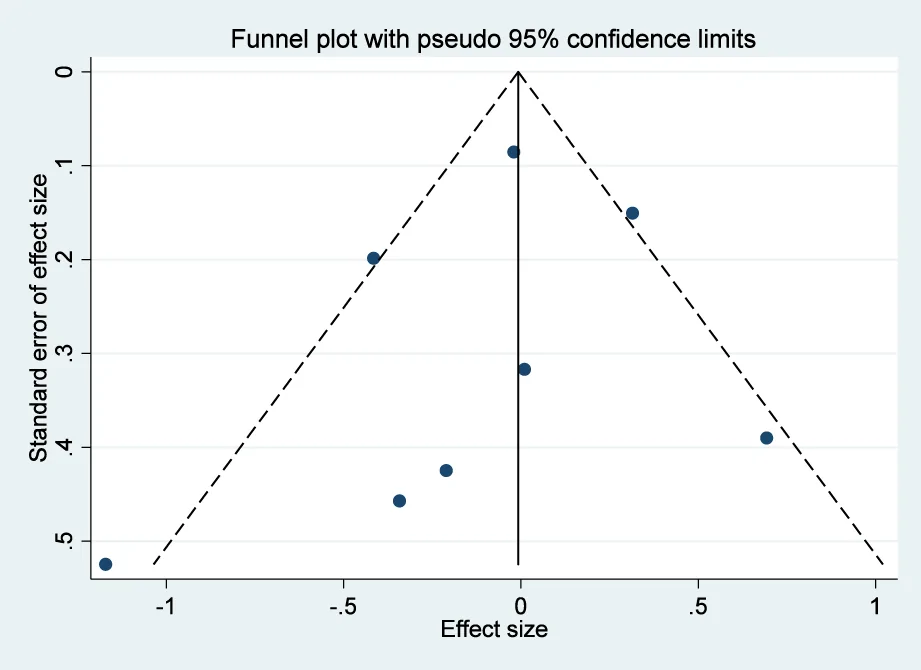
Funnel plot of the association between intraoperative use of dexmedetomidine and overall survival in cancer patients after surgery.

**FIGURE 7:**
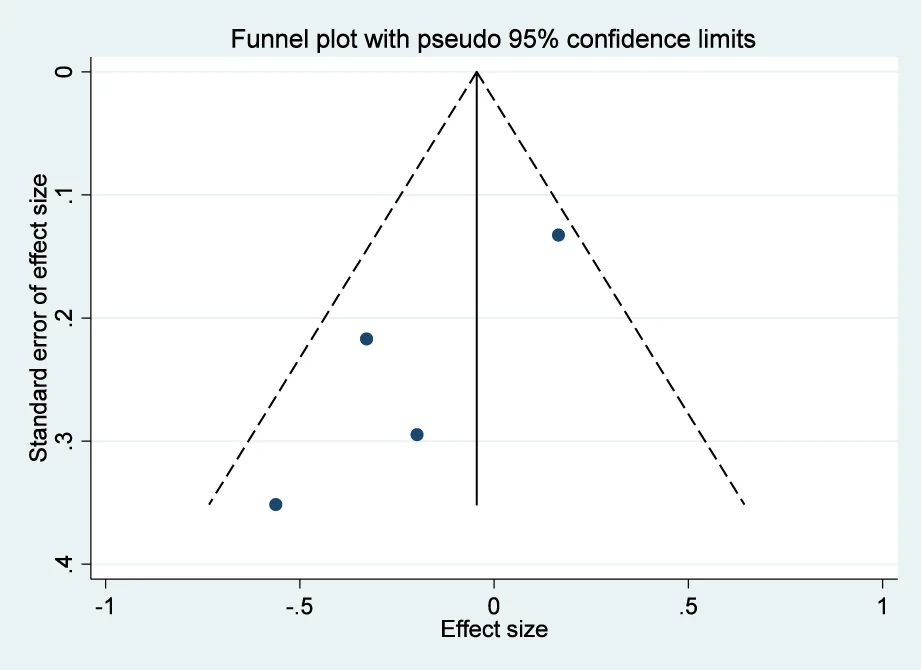
Funnel plot of the association between intraoperative use of dexmedetomidine and recurrence-free survival in cancer patients after surgery.

Funnel plots suggested some asymmetry, raising the possibility of publication bias (Figures 6, 7). However, Begg’s test (OS: *P* = 0.174; RFS: *P* = 0.308) and Egger’s test (OS: *P* = 0.571; RFS: *P* = 0.098) did not provide statistical evidence of publication bias.

## Discussion

In this meta-analysis of eight real-world studies encompassing 4,613 cancer surgery patients, we found that intraoperative dexmedetomidine use was not statistically significantly associated with either OS or RFS. Although a potential survival benefit emerged in the colorectal cancer subgroup, the overall evidence does not support routine dexmedetomidine administration for the purpose of improving long-term oncological outcomes.

The impact of dexmedetomidine on postoperative prognosis in cancer patients has long been a contentious issue in perioperative anaesthesia management. Some preclinical studies suggest that the drug may indirectly suppress tumour growth by mitigating surgical-stress-induced immunosuppression and reducing inflammatory cytokine release (Xu et al., 2025; Jun et al., 2023). Moreover, dexmedetomidine reduces intraoperative opioid requirements, and opioids have been shown to impair cellular immunity and promote tumour angiogenesis (Løhre et al., 2023; Omokore et al., 2025; Zhou et al., 2025; Gi et al., 2022; Wang et al., 2024). On this mechanistic basis, a survival benefit has been hypothesised. However, our overall findings do not support this hypothesis, aligning with recent meta-analyses of randomised controlled trials (Zhang et al., 2025). Nevertheless, individual studies have reported discrepant results. For instance, Cata et al. (Cata et al., 2017) found that intraoperative dexmedetomidine was significantly associated with worse OS, but not RFS, in non-small-cell lung cancer patients, suggesting potential tumour-promoting effects via α2-adrenoceptor activation, HIF-1α and VEGF upregulation, or immunosuppression (Cata et al., 2017). Similarly, Lavon et al. (Lavon et al., 2018) demonstrated in animal models that dexmedetomidine promoted metastasis of breast, lung, and colon cancer. In contrast, Ren et al. (Ren et al., 2024) reported significantly better OS in the dexmedetomidine group, with multivariate analysis identifying dexmedetomidine as an independent protective factor. Zha et al. (Zhao et al., 2021) observed that dexmedetomidine enhanced renal cancer cell proliferation and invasion *in vitro*, yet their clinical retrospective analysis found no survival difference, implying that host immune and systemic factors may offset any pro-tumour effects. These differences may stem from variations in the types of studies included, tumor types, sample sizes, follow-up periods, and the levels of control over confounding factors.

The most notable subgroup finding of this study is that intraoperative dexmedetomidine use was significantly associated with improved OS in patients with colorectal cancer. Although the effect size is considerable, this result warrants cautious interpretation. First, this subgroup comprised only two retrospective studies with a limited combined sample size (approximately 1,000 cases), which may introduce unmeasured confounding biases. Second, colorectal cancer might be particularly sensitive to the biological actions of dexmedetomidine, though the precise mechanism remains unclear; it has been speculated that this sensitivity could be related to the expression level of α_2_-adrenergic receptors within the colorectal tumor microenvironment and the activation state of downstream signalling cascades, such as PI3K/Akt and MAPK. Importantly, our subgroup finding of a significant OS benefit in colorectal cancer appears to conflict with the preclinical evidence from Lavon et al. (Lavon et al., 2018), which showed increased metastasis in colon cancer mouse models. We believe this apparent contradiction does not necessarily reflect a genuine discordance, but rather highlights fundamental differences between preclinical models and real-world clinical settings. Animal models typically employ immunodeficient or syngeneic mice with tumour cells injected subcutaneously or intravenously, which cannot fully recapitulate the complex tumour microenvironment, surgical stress responses, and postoperative immune reconstitution experienced by human colorectal cancer patients undergoing curative resection. However, direct translational evidence to support this hypothesis is currently lacking. Therefore, the colorectal cancer subgroup result should be regarded as hypothesis-generating rather than confirmatory, and requires prospective validation. In contrast, no significant associations were observed for renal cell carcinoma or other tumour types (lung, liver, breast, peritoneal). Subgroup analyses by region (Asian vs. non-Asian) and sample size (>500 vs. ≤500) also yielded negative results. Notably, the pooled HR for non-Asian populations tended toward an increased risk (HR = 1.24), though not statistically significant, suggesting possible population heterogeneity that may relate to differences in tumour biology, perioperative management (e.g., dosing, timing, and combination regimens), or genetic background.

From a clinical decision-making perspective, the current evidence does not support the selective use or avoidance of dexmedetomidine with the sole aim of improving long-term oncological outcomes. When interpreting these results, clinicians should recognise that the exposure measured was primarily intraoperative administration; postoperative use was not systematically documented. Thus, any clinical recommendation should consider the total perioperative exposure rather than intraoperative use alone. For cancer surgery patients who require dexmedetomidine for its sedative, analgesic, or sympatholytic effects, it can still be used according to conventional indications without undue concern about negative survival impacts. However, for colorectal cancer patients, the positive signal, though preliminary, warrants attention. Future rigorous multicenter prospective cohort studies or pragmatic RCTs, with dexmedetomidine use as the grouping variable and OS/RFS as primary endpoints, are warranted. In addition, future research should focus on the following aspects: 1) standardising the administration of dexmedetomidine (dose, timing, duration), and performing dose-response analyses; 2) collecting and adjusting for key confounders, including tumor stage, surgical stress indicators (such as intraoperative blood loss, transfusion status), inflammatory markers (e.g., IL-6, CRP), and cumulative opioid dosage; 3) conducting biomarker-driven subgroup analyses; and 4) exploring potential interactions with chemotherapy or immunotherapy, as perioperative use may influence the efficacy of subsequent adjuvant therapy.

Several limitations of this study should be acknowledged. First, because all included studies were retrospective observational designs, the pooled results reflect statistical associations rather than causal relationships. Although most studies performed multivariable adjustments, residual confounding and selection bias cannot be entirely excluded. Therefore, our conclusions should be viewed as hypothesis-generating, and prospective studies or large-scale randomised trials are needed to validate any potential causal effects. Second, most studies did not report median follow-up duration; differences in follow-up length may affect the estimation accuracy of OS and RFS. Third, funnel plot asymmetry suggested possible publication bias, although Begg’s and Egger’s tests did not reach statistical significance. Given the small number of included studies and low test power, the possibility of unpublished negative results cannot be ruled out. Fourth, there was considerable heterogeneity in dexmedetomidine dosing regimens (loading dose, maintenance rate, infusion duration) across studies, precluding dose-effect analyses. This regimen heterogeneity not only contributes to statistical heterogeneity but may also introduce residual confounding. Fifth, our exposure definition was limited to intraoperative administration; some patients may have received dexmedetomidine postoperatively via patient-controlled analgesia or multimodal analgesia, which was not separately reported in the original studies. Thus, the observed effects may reflect total perioperative exposure rather than intraoperative use alone. Sixth, dexmedetomidine administration was determined by clinical anaesthesiologists based on clinical judgment, not random assignment. Patients receiving dexmedetomidine may differ systematically from those not receiving it—they may have higher perioperative cardiovascular risk, require deeper sedation, or undergo more complex surgeries; conversely, dexmedetomidine may have been avoided in patients with haemodynamic instability or bradycardia. Even after adjustment in multivariable models, such baseline differences inevitably introduce residual confounding that cannot be fully eliminated by statistical methods. Therefore, the observed associations should not be interpreted as causal, and the pooled HR estimates may themselves be biased by non-random treatment allocation.

## Conclusion

In summary, this meta-analysis of real-world evidence suggests that intraoperative dexmedetomidine use is not statistically significantly associated with overall survival or recurrence-free survival in cancer patients. The potential survival benefit observed in the colorectal cancer subgroup requires further verification. Current evidence does not support routine dexmedetomidine use for the purpose of improving long-term prognosis, nor does it permit causal inferences. Future large-sample, prospective, well-controlled real-world studies, combined with biomarker analyses, are needed to clarify the role of dexmedetomidine in specific tumour types or patient subgroups.

## Data Availability

The datasets presented in this study can be found in online repositories. The names of the repository/repositories and accession number(s) can be found in the article/supplementary material.
